# Does political conflict tilt finance-renewable energy dynamics in Africa? Accounting for the multi-dimensional approach to financial development and threshold effect of political conflict

**DOI:** 10.1016/j.heliyon.2023.e14155

**Published:** 2023-03-01

**Authors:** Obumneke Bob Muoneke, Kingsley Ikechukwu Okere, Obiamaka Priscilla Egbo

**Affiliations:** a FITC Nigeria, Murtala Muhammed Way, Ebute-Metta, Lagos, Nigeria; b Department of Finance, University of Lagos, Akoka-Yaba, Lagos State, Nigeria; c Department of Economics, Banking and Finance, Gregory University, Uturu, Abia State, Nigeria; d Entrepreneurial /Skills Development Centre/Enterprise Venture, Gregory University, Uturu, Abia State, Nigeria; e Department of Banking and Finance, University of Nigeria, Enugu Campus, Enugu State, Nigeria

**Keywords:** Political conflict, Finance, IV-GMM, Renewable energy, Africa

## Abstract

In order to shed empirical light on the impact of the multi-dimensional decomposition of financial development indicators on renewable energy usage, this study investigates the threshold effect of political conflict on finance-energy dynamics in Africa. The research output relies on a panel of 46 African nations from 2010 to 2020, using IV-GMM estimators that are robust to cross-sectional dependence and allow for heterogeneous slope coefficients. The results of direct, indirect, and threshold equations show that i.) Financial development indicators spur renewable energy consumption, while political conflict drags it. ii.) There is a threshold at which financial development could spur renewable energy in some regions of Africa, and the tendency of financial development to maintain such capacity is conditioned on the accessibility of financial facilities and political conflict/stability within a specific range of threshold values. iii.) the threshold level assessment shows that 11 countries in the panel, including Botswana, Mauritius, Cape Verde, Namibia, Seychelles, Zambia, Sao Tome and Principe, Gabon, Ghana and Benin Rep., are above the threshold level of political conflict in Africa. From a policy angle, driving up renewable energy consumption in Africa requires the government to provide enabling safety net environment for the diversification of finance options targeting innovative shifts away from traditional energy sources to the expansion of alternative renewable energy ventures.

## 1 Introduction

The finance-renewable energy nexus has been investigated by a multitude of scholars, with most of the studies examining the direct relationship between financial development and renewable energy consumption in a host of political and economic jurisdictions [[1], [2], [3]]. However, the topic remains evergreen as the financing gap remains a standout challenge to developing countries, especially African countries, in the proposed transition to cleaner forms of energy. The problem is diverse in the African scenario in that most countries still have less-developed and developing capital markets alongside bank financial institutions with limited bank capitalisation compared to sister banks in the various global financial centres in the Western world. Furthermore, limited fiscal budgets to cover capital expenditure inclusive of green infrastructures from most countries on the African continent worsens the financing gap, leaving room for rich polluting countries in Asia, America, and Europe to contribute their quotas financially in that regard [4]. posited that local authorities’ attitudes were rather careful, caused by the financial constraints of local budgets and the scope of obligatory tasks, which made renewable energy investments, not the most urgent. However, public aid can be a distinguishing factor in increasing renewable energy investments.

Following ABSA Africa Financial Markets Index 2022 report, South Africa, Mauritius, and Nigeria ranked 1–3, respectively. A host of eastern African countries (Uganda, Botswana, Namibia & Kenya) occupies 4th, 5th, 6th & 8th, and two North African countries (Morocco & Egypt) occupy 9th and 10th on the most developed financial markets in Africa for 2022. Despite recorded progress in capital market development for the countries mentioned above, the domestic capacity to finance renewable energy technologies still needs to be improved. The current look at Africa's current energy generation mix as of 2020 shows that all major fossil fuels occupy a combined 90.5% of total energy generated, leaving RE sources such as wind, solar, bioenergy, nuclear, and hydropower to the remaining 9.5% (BP Energy Outlook, 2020). Post COP-26 and COP-27, policymakers of African origin have been explicit about Africa's disadvantage regarding required funding to transition to cleaner forms of energy in line with the 2030 and 2050 climate targets. Excerpts from the African Energy Outlook 2019 report show that a cumulative investment of 2.6 trillion dollars in energy is required between 2019 and 2040 to provide accessible energy to Africans [5]. There are several financial pledges from the private capital market alliances, EU, EU member states, Bretton Woods institutions, and high-emitting nations towards renewable energy investment in Africa.

Amidst the gap, there has been noticeable adoption of renewable energy technologies in North Africa. The evidence of the said adoption translates to the citing of the largest solar power plant worldwide with 580 MW capacity in Morocco and the future target by the Kingdom of Morocco to generate 52% of its electricity from renewable energy sources by 2030 [6]. However, Southern Africa supersedes Northern and Eastern Africa in current capacity by 11,000 MW and 14,000 MW, respectively. Many renewable projects are still ongoing in Southern, Western, Central, and Eastern Africa, which may be susceptible to several challenges impeding their completion. Several factors pencilled to be one of the challenges facing the adoption of RETs in African countries, including limited access to credit facilities for renewable energy producers, higher upfront costs, political propaganda, favourable political treatment for fossil fuel options, greater information costs to investors due to a less-developed financial sector and choice of less-risky projects such as fossil fuel projects ([[7], [8], [9], [10], [11]]).

The state of academic literature is bereft of geopolitics (conflict) and its impact on the finance-renewable energy nexus in the case of Africa. The first group of scholars concentrated on renewable energy and financial development nexus in Africa and other regions of the world ([2,3,[12], [13], [14], [15], [16], [17], [18], [19]]). The second group of scholars investigated the finance-renewable energy nexus embossing the moderating role of institutional quality ([[20], [21], [22], [23]]). Acknowledging the role of finance in increasing the adoption of RETs in Africa, it is pertinent to note that certain political factors (wars, ethnic clashes, militias, and acts of terrorism) may deter the level of renewable energy investment or disrupt ongoing renewable energy projects. Furthermore, renewable energy reduces the rate of international conflicts and geopolitical risks associated with the energy transition ([24,25]). The expected theoretical expectation and empirical posits from OECD-based studies may be erroneous in the case of Africa due to the dynamics of conflict on the continent. The aura for financing and installing RET in Africa is distraught by unending political instability, pastoralist-nomads conflict in the Sahel, religion-induced terrorism, secessionist agenda, multiplier effects of climate change, land grabbing, age-long inequality, and energy insecurity in impoverished areas. Sustained conflicts are rampant in the Sahelian region and the horn of Africa. Recently, acts of terrorism, farmers-herders conflicts, and frequent government takeovers by the military are the present realities in West and Central Africa. A United States Institute of Peace's special report posited that the gains of renewable energy are yet to be experienced by South Sudanese residents and other conflict settings.

Conflicts create an uneven distribution of renewable energy projects amongst crisis-free and crisis-laden regions within the African continent, which further worsens energy insecurity amidst the proposed renewable energy transition. The case of renewed conflict may arise, as documented by Ref. [25], where the energy transition to renewable energy causes energy-related conflict. Dissecting the varieties of renewable energy, hydropower dams & reservoirs, concentrated solar power farms and bio-fuel plantations require a relatively higher landmass which may exacerbate armed conflict with rival rebel groups within the Sahara and Sahelian regions. A supporting USAID report in 2010 shows that planning and construction of hydroelectric dams are associated with increased tensions in Lesotho, Sudan, and Uganda. Furthermore, a plethora of empirical studies considering geopolitical risks are conducted outside Africa, where there is relative peace, absence of wars, ethnic clashes, terrorism, and political instability ([[26], [27], [28]]). The aforementioned necessitates the decision by the authors to investigate the role of political conflict in the finance-renewable energy nexus for African countries.

The study intends to contribute to the knowledge repository stated thus; (i) ascertain the impacts of financial development and political conflict on renewable energy consumption in Africa; (ii) establish the moderating effect of political conflict on the finance-energy nexus in Africa, and (iii) establish the level of political conflict threshold that would be scaled up to triggers the financial system to improve the level renewable energy consumption in Africa. Existing studies mainly concentrated on the effect of a single variable as a proxy for financial development on renewable energy consumption. These studies used domestic credit to the private sector [12], various components of financial development ([2,3,[13], [14], [15], [16], [17], [18], [19]]) among others. This means that the extant literature is somewhat biased against the effect of global financial development indicators, notwithstanding that there are many indicators of financial development indexes as proposed by the IMF. Furthermore, past studies have only examined the moderating role of institutional quality ([[20], [21], [22], [23]]). What remains unclear, particularly in Africa, is i.) how political conflict moderates the effect of IMF multidimensional financial development on renewable energy. ii.) the threshold value that could harm or trigger financial development on demand for renewable energy consumption in Africa and countries that fall below and above the threshold of political conflict. There is a dearth of empirical exposition yet to articulate the roles of political conflict in the finance-renewable energy nexus. There is also a need for extensive investigation of how the IMF's multidimensional financial development interacts with political conflict to influence RET in Africa, which will significantly enrich the robustness of the existing evidence about the finance-energy nexus in Africa. Thus, this study will provide information to aid evidence-based policies in Africa. Finally, on the methodological front, this study adopted Instrumental Generalize Methods of Moment (GMM). As a robust model, IV-GMM accounts for variable omission bias and produces consistent estimates, robust in the presence of endogeneity, omitted variable(s) bias, and auto-correlation [29] and efficient results in the presence of unknown heteroscedasticity vis-a-vis its orthogonality condition. The chosen method contributes to knowledge addendum due to the novelty of our methodological approach and the scarcity of authors using the aforementioned methods in investigating the finance-renewable energy relationship in Africa.

The following section 2 builds on the already established premise above by reviewing the relevant empirical studies and providing theoretical context, while the following section 3 explains our research methodology and the variables and data sources we used for our analysis. Results are presented and discussed in Section 4, and conclusion and policy implications are presented in Section 5.

## 2 Literature review

### 2.1 Theoretical orientation

The foundational theoretical link underpinning this empirical adventure lies in the financial development and energy consumption nexus [30]. posited that financial development has three effects on energy use namely; a) direct effect, b) business effect c) wealth effect. The direct effect mirrors a financial development scenery where consumers access loans at a cost-competitive rate to purchase energy-intensive consumer goods. The business effect through the auspices of high-level financial development translates to firms’ ability to access low-interest loans easily. Finally, the wealth effect surfaces when increased economic confidence leads to economic expansion and promotes energy demand. The mainstay is to ensure that energy demand is met with more renewable energy sources rather than non-renewables for firms or households. Focusing on the finance-renewable energy link, financial development impacts renewable energy demand through the auspices of sophisticated financial institutions and high-value capital markets funding for green renewable energy projects. Furthermore, incentives such as ease and cost-efficiency to firms and households are offshoots of a developed financial system. Therefore, it guarantees low-cost financing to green-compliant economic units and their environmental-friendly business activities ([1,31]).

Besides financing, several barriers to renewable energy investment in Africa are classified under geopolitical risks. There are two causal pathways visible in geopolitical risks and renewable energy nexus. Firstly, geopolitical risks play a massive role in the advent of renewable energy through the auspices of energy security, which, most times, form the fundamental root cause of conflict. In scenarios of energy insecurity, countries may hasten their renewable energy transition to eradicate energy poverty in an impoverished region [24]. On the other hand, renewable energy provides some opportunities to terrorists and is helpful to them in cooking and heating in isolated areas away from intense security surveillance. Those mentioned above decrease their dependence on fossil fuels and guarantee their energy autonomy [32]. In other cases, conflict-torn areas may also be hostile to installing renewable energy technologies and green investors. Secondly, renewable energy has a significant negative impact on geopolitical risks driven by global economic growth, rising fossil fuel prices, and technological innovations. Depending on the country's context, the geopolitical risk may reduce renewable energy consumption, or renewable energy production may reduce geopolitical risks.

### 2.2 Empirical review

The structure of this section is divided into three key themes central to the objectives of this study, namely; a) financial development-renewable energy nexus, b) political conflict-renewable energy nexus c) other factors that influence the finance-renewable energy nexus.

### 2.3 Financial development and renewable energy nexus

The primary theoretical link that supports the authors' empirical adventure lies in the fact that financial development affects the environment through the auspices of energy consumption ([30,33]). By extension, our study's central focus rests on renewable energy use as a subset of energy use [14]. document that improved financial development, human capital, and renewable energy electricity guarantees lesser carbon emissions and a sustainable environment. Demystifying financial development into its broad components, bank-based and stock-based financial development [13], pulled an important finding that an increase in bank-based financial development does not lead to a proportionate increase in renewable energy consumption in the United States.

Contrary to the above discussion, renewable energy adoption in the United States is boosted by a wonderful increase in the stock market and the overall financial development index. Using a panel fixed effects model [1], dissected financial growth into the banking industry, the bond market, and the capital market, and then regressed that growth on renewable energy use. The results showed that all three initiatives had a favourable effect on the amount of renewable energy used by EU countries. New EU members' renewable energy use was unaffected by the growth of capital markets. In contrast, writers like [34] have argued that bank measures of financial development are more important than stock market index measurements [35]. argued that it is necessary to include stock market capitalization as a measure of financial progress. The case takes another nosedive in the study of [36]. Using the ARDL-PMG econometric technique, the study found that financial development reduces renewable energy consumption for western China and China in the long-run. Chinese efforts to enhance their use of renewable energy sources are hampered by the country's underdeveloped financial sector and a sluggish stock market.

Another study by Ref. [12] examined the impact of financial development and renewable energy on environmental quality in the Middle East and North Africa region using domestic loans to the private sector as a measure. Their findings depict a weak influence of financial development and renewable energy consumption on environmental quality, necessitating policymakers' attention to developing the financial system architecture to support the adoption of massive renewable energy technologies in MENA region [3]. also adopted domestic credit provided by the financial sector and domestic credit to the private sector to measure financial development. The critique behind adopting these measures lies in the fact that both measures are obtainable in non-renewable energy investment. Therefore, finance wields a positive and weak effect on energy consumption (renewable energy, to be precise).

A practical example is seen in Ref. [37] as one of the several studies that adopted the domestic credit to the private sector by banks measure. The study found that an increase in the level of financial development prompts a spike in renewable energy consumption [15]. using system-GMM, investigated the financial development, renewable energy use, and carbon emission nexus in the case of sub-Saharan Africa [15]. unlike studies such as [3,12,17,37] adopted a broader measure of financial development which encompasses the access, depth, and efficiency. The study found that financial development and its sub-components contribute to environmental pollution in sub-Saharan Africa. On the other hand, financial market development has fewer adverse environmental effects compared to the former. Another study conducted by Ref. [16] stood out amongst the park deploying a more comprehensive measure of the financial development index provided by the IMF in investigating the effect of economic policy uncertainty and financial development on renewable energy consumption in Brazil, Russia, India, China, South Africa, and Turkey. The authors found that FD, environmental regulations, and shadow economy spurs renewable energy use in BRICST countries.

Evidence from West Africa on finance-renewable energy nexus resides in the study of [17]. [17] adopted domestic credit to the private sector as a measure alongside a plethora of econometric techniques such as FMOLS, DOLS, and Pooled Mean Group estimators [17]. posited that financial-related reforms exacerbated environmental pollution in the West African region, while renewable energy demand is favourable to the environmental health of the West African region [18]. also adopted the broader measure of financial development as seen in Refs. [[15], [16], [17]] and reaffirmed status quo that finance spurs renewable energy consumption in Nigeria. Another study by Ref. [2] utilized several variables from the WDI dataset to measure financial access and depth, distinguishing itself from studies that applied the IMF variety. Using the two-step system GMM, we find that in high-income nations, financial development has a favourable effect on the use of renewable energy, whereas in low- and middle-income countries, it appears to be unimportant.

The next strand of literature relates to the finance-renewable energy nexus and the role of institutional quality. Research conducted in Tunisia by Ref. [38] found that renewable energy use is bolstered by high quality institutions and is hindered by financial development [22]. adopted the coupling and decoupling effect approach in studying the role of governance in the finance-renewable energy nexus for sub-Saharan Africa. Findings show that a 1% increase in the coupling effect of renewable energy and governance exacerbates climate change effects by 0.79%. On the other hand, the decoupling effect revealed that governance, FDI, and income level exacerbate climate change. At the same time, renewable energy consumption lessens climate change and its devastating impact in the case of sub-Saharan Africa.

### 2.4 Political conflict and finance-renewable energy nexus

This section features studies conducted on the geopolitical risks and renewable energy nexus across political and economic jurisdictions. Several studies on the finance-renewable energy nexus eluded the probability of conflict disrupting the progress of energy transition [39]. affirm that the triangular matrix of energy, environment, and conflict has yet to receive much attention in the research arena. However, there is the existence of empirical studies on geopolitical risks and environmental quality [40]. adopted terrorism as a measure of geopolitical risk in a bid to analyse the impact of terrorism on environmental quality in Afghanistan, Iraq, Nigeria, Philippines, Pakistan, Syria, Thailand, and Yemen. Results depict that terrorism worsens environmental degradation in the selected countries. Another supporting study justifying the aim of this study resides in the empirical effort of [41] where the relationship between climate change, conflicts, and political instability in MENA countries was subjected to a causality analysis. Results show a unidirectional causality from political instability to climate change in the MENA region, in line with [42] stating that political instability contributes to the high level of environmental degradation in sub-Saharan Africa. Another MENA region-based study conducted by Ref. [26] found that political stability is necessary for renewable energy development in the region.

In the opposite direction [43], adopted a distinct econometric technique (time-varying parameter Bayesian vector autoregressive model to investigate the dynamic interactions between geo-political risk (GPR) and renewable energy consumption growth (RECG). Findings show that RECG shocks decrease geo-political risks throughout the sample period. Another exciting finding increasing the vertices of academic literature resides in the study of [27] for 32 OECD countries. Findings show that economic globalisation and political risk reduce renewable energy consumption in 32 OECD countries. Most studies incorporating geo-political risks in the renewable energy discourse are conducted outside Africa. Only some studies are focused on Middle East and North African countries, which do not cover the major conflict-torn areas on the African continent, thereby making our empirical input significant.

## 3 Data and methodology

### 3.1 Data sources

Based on the combination of cross-sectional and time series data of 46 chosen countries in Africa nations from 2010 to 2020. These countries see (Appendix 5) were chosen because they have the most comprehensive data sets. This dataset has a total of 460 observations. In light of this, we can conclude that the panel data consists of N > T, that is, the number of cross-section is greater than the number time period. The main objective is to estimate the threshold effect of political conflict on finance-renewable energy dynamics in Africa. Table 1 highlights the data sources used for this analysis and exposition.

**Table 1:** Descriptive statistics (panel A) and correlation matrix (panel B) 2010–2020.

Panel A	Fin1	Fin2	Fin3	Fin4	Fin5	Fin6	Fin7	Fin8	Fin9	Gdp	Poptol	Ren	Trade	Confl
Mean	0.151	0.127	0.120	0.498	0.235	0.068	0.071	0.046	0.062	3.135	6.883	1.707	1.824	−0.524
Median	0.110	0.071	0.062	0.506	0.192	0.001	0.016	0.022	0.009	3.070	4.041	1.876	1.808	−0.426
Maximum	0.643	0.899	0.878	0.784	0.731	0.915	0.789	0.992	0.535	4.202	8.322	1.987	2.335	1.111
Minimum	0.03	0.00	0.00	0.11	0.05	0.00	0.00	0.00	0.00	2.43	4.94	−0.149	1.32	−2.699
Std. Dev.	0.119	0.163	0.174	0.140	0.142	0.160	0.126	0.152	0.109	0.417	0.700	0.374	0.187	0.827
Skewness	2.227	2.473	3.145	−0.435	1.998	2.527	3.305	4.881	2.259	0.752	−0.532	−2.570	0.215	−0.285
Kurtosis	7.82	9.20	12.80	3.66	6.48	8.52	15.87	28.57	7.75	2.73	3.02	10.27	2.87	2.67
Jarque-Bera	907.795	1325.444	2858.203	18.406	591.614	1181.149	4414.566	15791.860	906.271	49.163	23.890	1672.330	4.295	9.158
Probability	0.000	0.000	0.000	0.000	0.000	0.000	0.000	0.000	0.000	0.000	0.000	0.000	0.017	0.010
Panel B														
Fin1	1.000													
Fin2	0.712	1.000												
Fin3	0.883	0.508	1.000											
Fin4	0.509	0.280	0.309	1.000										
Fin5	0.945	0.830	0.856	0.555	1.000									
Fin6	0.582	0.245	0.371	0.256	0.381	1.000								
Fin7	0.745	0.417	0.668	0.279	0.629	0.387	1.000							
Fin8	0.684	0.307	0.685	0.275	0.582	0.237	0.341	1.000						
Fin9	0.903	0.436	0.768	0.364	0.712	0.746	0.772	0.701	1.000					
Gdp	0.485	0.478	0.294	0.336	0.475	0.195	0.465	0.259	0.416	1.000				
Poptol	−0.111	−0.3403	0.026	−0.046	−0.165	0.116	−0.094	−0.071	−0.022	−0.429	1.000			
Ren	0.495	0.613	0.307	0.251	0.527	0.082	0.584	0.162	0.369	0.732	0.521	1.000		
Trade	0.167	0.253	0.140	0.023	0.204	−0.049	0.145	0.126	0.091	0.41	−0.540	−0.468	1.000	
Confl	−0.319	−0.348	−0.274	−0.216	−0.370	−0.0351	0.297	0.188	0.198	0.528	−0.5547	−0.4767	0.494	1.000

### 3.2 Model building

The modeling strategy is based past empirical narration of [20,[39], [40], [41], [42],^44^].

This study presents a dynamic panel model in the form:

(1) $${ren}_{it}=\alpha_{i}+{\beta Fin}_{it}+{\emptyset confl}_{it}+{\gamma Z}_{it}+\varepsilon_{it}$$

The eqt. (1) depicts the direct impact of financial development and political conflict on renewable energy consumption, the parameters estimates and their respective a priori expectations are β>0,∅<0,andγ>0. ${ren}_{it}$ is renewable energy use, i in time t ; ${Fin}_{it}$ vector of multi-dimensional decomposition of financial development index; ${confl}_{it}$ is the political conflict; Z denotes a vector of other control variables (Gdp, Population Total and Trade openness); ε captures the stochastic error term. The second objective shows the impact of the interactive term between political conflict and financial development as closely implemented by Ref. [20]. Thus our eqt 1 is extended as thus;

(2) $${ren}_{it}=\alpha_{i}+{\beta Fin}_{it}+{\emptyset confl}_{it}+\varphi \left({\mathrm{Int}}_{it}\right){+\gamma Z}_{it}+\varepsilon_{it\;}$$

Where ${\mathrm{Int}}_{it}$ is the interaction term and is given as thus: ${Fin}_{it}*{Confl}_{it}$. Eqt.2 above defines ${\mathrm{Int}}_{it}\;as$ the moderating role of political conflict on finance-renewable energy nexus in Africa. The net effect of financial development is accounted for by accessing the differential effect from eqt 2. as thus;

(3) $$\frac{{\partial ren}_{it}}{\partial {Fin}_{it}}=\beta +{\varphi confl}_{it}$$

Where β is the direct effect of financial development index, φ is the total effect.^1^

The coefficients of β and φ in the eqt.3 shows the moderating role of political conflict on the finance-energy relationship. Similarly to Ref. [45] the coefficients of β and φ can be interpreted as thus; in a situation where β > 0 & φ < 0, it shows that the direct effect of Fin drives renewable energy consumption, and the total effect drags the positive effect of Fin; If β < 0 & φ > 0, it implies that the direct effect of Fin reduces renewable energy consumption, and the total effect mitigates the negative of effect Fin.

#### 3.2.1 The threshold of political conflict is expressed as thus

(4) $$ren=\frac{\beta }{\varphi }$$

The condition in eqt.4 holds if the marginal effect ME < 0 as clearly shown in eqt.5 below:

(5) $$ME<0\Rightarrow \left[\begin{array}{c} confl<{Thres.}_{confl},for{Thres.}_{confl}=\left(-\frac{\beta }{\varphi }\right)and\;\varphi >0\\ confl>{Thres.}_{confl},for{Thres.}_{confl}=\left(-\frac{\beta }{\varphi }\right)and\;\varphi <0 \end{array}\right]$$

Where: ${Thres.}_{confl}$ implies the threshold, ME is the marginal effect of value of political conflict.

### 3.3 Estimation technique

Using the IV-GMM, parameter estimation is handled. Endogeneity makes it possible for conventional estimating techniques, such as Ordinary Least Squares (OLS), to produce erroneous findings when applied to estimate the aforementioned equation (2). In light of this, the IV-GMM served as the principal estimation method in this investigation. The IV-GMM efficiently handles endogeneity sources such as reverse causality, measurement error, and variable omission bias, and delivers trustworthy estimates. In addition, since the IV-GMM applies the orthogonality condition, it is resistant to autocorrelation and yields trustworthy results when heteroscedasticity is unknown [46]. This study employs the Kleibergen-Paap Wald F statistic, Hansen test statistics, and Kleibergen-Paap Lagrange Multiplier to evaluate the validity and dependability of the IV-GMM results. Multiple recent research employed the IV-GMM estimator ([19,33]).

### 3.4 Empirical results

#### 3.4.1 Preliminaries

Table 1 displays the raw data's descriptive statistics and correlation matrices, which shed light on the shared properties of the study's variables. According to the pattern, the variables' mean values are higher than their medians. Inferring from the preceding result, the distributions of the variables are not symmetrical; some are skewed to the right, while others are biased to the left. To check if the mean is an appropriate representation of the data, the standard deviation is also calculated and compared with the mean. Low standard deviation coefficients indicate that the data tend to be concentrated near the mean. There is no statistically significant deviation between the data and the mean, suggesting that the mean values reflect the data. Multiple indices of political conflict show that, on average, the level of political conflict in Africa is quite low, and their minimum and maximum values support this conclusion. Between negative 2.5 and positive 2.5. In conclusion, the political war in Africa is going at a snail's pace compared to conflicts in other countries. Because of this, a political framework may emerge to direct financial arrangements, which may enhance or limit the use of renewable energy. Outside of GDP, Trade, and Conflict, all other variables exhibit a leptokurtic (>3) data distribution. The degree of linear association between the variables is displayed in the correlation matrix, which can be seen in the lower portion of Table 1 -Panel B. If there is a linear and inverse link between renewable energy use and the other factors, then all of them except political conflict have positive correlations. The positive coefficients of correlation between renewable energy and all other variables are indicative of a linear relationship. These connections stress the importance of conducting additional research with more cutting-edge methods.

### 3.5 Presentation and discussion of results

Table 2 reports the results of the [47] test for cross-sectional dependence. The test statistics reject the null hypothesis of cross-sectional independence for Fin1-9, logGdp, logPoptol, logRen, logTrade Confl, suggesting that the data series are cross-sectionally dependent.

**Table 2:** Pasaran (2021) Cross-section dependence test.

Variable	CD-test	p-value	average joint T	mean ρ	mean abs(ρ)
Fin1	13.753	0.0000	11	0.130	0.440
Fin2	50.825	0.0000	11	0.480	0.570
Fin3	18.101	0.0000	11	0.170	0.480
Fin4	2.203	0.0280	11	0.120	0.340
Fin5	13.785	0.0000	11	0.130	0.460
Fin6	1.680	0.0030	11	0.220	0.090
Fin7	1.531	0.0260	11	0.310	0.310
Fin8	0.935	0.0500	11	0.110	0.430
Fin9	−0.122	0.0030	11	0.000	0.290
logGdp	25.021	0.0000	11	0.230	0.610
logPoptol	95.738	0.0000	11	0.900	0.900
logRen	21.085	0.0000	11	0.200	0.560
logTrade	9.978	0.0000	11	0.090	0.450
Confl	1.502	0.0330	11	0.010	0.400

Notes: Under the null hypothesis of cross-section independence, CD ∼ N(0,1) P-values close to zero indicate data are correlated across panel groups.

Appendix 2 reports IV-GMM estimates following Eq. (2). Columns [1,5,9] relate to the baseline model as the financial development, institution, and market index, respectively. Columns [2], [3], [4], [6], [7], [8] report estimation on the multi-dimensional components of financial institutions and market index, comprising of access, depth, and efficiency index, respectively. In all the Column [[1], [2], [3], [4], [5], [6], [7], [8], [9]], the political stability index and its interaction with the baseline model and the multi-dimensional approach to financial development index variable are introduced accordingly, while control variables-gdp per capita, population total, trade openness are incorporated in the specification to strengthen the analysis and mitigate error of omission of variable. This modeling approach enabled us to assess the consistency of the results. The diagnostic tests reported under the table using Kleibergen-Paap rk LM statistic, Cragg-Donald Wald F statistic, Hansen J statistic and Kleibergen-Paap rk Wald F statistic are robust and free from weak and invalid instrument. The R-squared, which captures the changes in the dependent variable as predicted by the independent variables, varies between 0.655 and 0.703.

The financial development index coefficient is positive and statistically significant at the 1% level across the nine Spec. (1–9). This finding is remarkable to some extent since it contradicts the axiom knowledge that most African economies have underdeveloped finance systems incapable of driving economic activities. For instance, in Spec.1-9, ceteris paribus, a 1% change in the financial development index may lead to a 0.013%–1.068% increase in renewable energy consumption in Africa. This finding reinforces the theoretical expectation of the finance-energy nexus as popularly reemphasised by Ref. [30] through a) direct effect, b) business effect c) wealth effect, and supports the rising empirical documentation of [[12], [13], [14], [15],^30^,^33^]) in the case of United States, Middle East, and North African region. Similarly [15], adopted a broader financial development measure, encompassing access, depth, and efficiency. The study found that financial development and its sub-components contribute to energy demand in sub-Saharan Africa. However, the result is also contrary to Ref. [2] findings that financial development exerts an insignificant impact on renewable consumption in low and middle-income countries even though it was positive. In the case of [36], the study found that financial development reduces renewable energy consumption for western China and China as a whole in the long-run. As expected, our measure of political conflict (confl) is negative and statistically significant across columns [[1], [2], [3], [4], [5], [6], [7], [8], [9]]. This finding supports the submissions by Ref. [40] in the case of Afghanistan, Iraq, Nigeria, Philippines, Pakistan, Syria, Thailand, and Yemen [41]; in the case of MENA countries. Similar to our first hypothesis, financial development and the political atmosphere both positively and negatively affect Africa's adoption of renewable energy sources.

The second objective of this study is to examine the interaction between financial development and political conflict. The political atmosphere could affect the financial institution and market depending on its nature. Political stability creates enabling environment for the financial system to foster appropriately and, as such moderate the number of credit facilities offered to firms and households. However, it may have a negative impact because it takes efforts and time to restore investors’ confidence after a series of political anomalies which are detrimental to financial development, especially firms and household that needs financial resources to acquire renewable energy. In this way, conflicts can create an uneven distribution of renewable energy projects amongst crisis-free and crisis-laden regions within the African continent, worsening energy insecurity in the face of the proposed transition to renewable energy. The extant literature suggests that one of the significant possible ways of understanding factors threatening the transition to renewable energy in Africa is to ensure that high levels of political stability are enthroned on the continent [25]. Therefore, we introduced the political conflict variable and its interaction (Int 1–6) with the financial development in Columns [[1], [2], [3], [4], [5], [6], [7], [8], [9]]; and examined the overall roles of political conflict in the relationship between financial development and renewable energy in Africa. This study analyses the conditional, unconditional, marginal impact and develops the threshold value of the political conflict. The sign of the interactive coefficients, such as φ in equation (3) is used. A negative coefficient implies that political conflict drags financial development on renewable energy consumption in Africa and vice versa. The unconditional effect (see Appendix 2) of financial development is positive in Columns [[1], [2], [3], [4], [5], [6], [7], [8], [9]] and ranges from 0.013 to 1.068, while the conditional (see Appendix 2) through the interaction of the political conflict with the financial development variable is also negative in Columns [[1], [2], [3], [4], [5], [6], [7], [8], [9]], ranges from (−6.821 to −0.52) and they are statistically significant at the 1% level (Appendix 2 & 3). We observed that the unconditional impact coefficients outweigh the conditional impact coefficient. The implication is that there is a positive marginal impact based on the average value of a political conflict, as shown in Appendix 3. Therefore, the marginal impact and threshold are computed using equation (4) and by making this equation (4) equal to zero ([44,45]). Based on this, the threshold value is equal to $\frac{\beta }{\varphi }$.These values are reported in Appendix 3.

To what extent political conflict in Africa could impede the growth of renewable energy is detailed in Appendix 3, along with the values at which this effect becomes discernible. The threshold values explored here varied between 0.002 and 1.396. The composite measure of financial development has a cutoff value of 1.396. The table shows that Fin5 (financial institution development) and Fin9 (financial market development) each need to be at least 0.181, while Fin6 (financial market development) has to be at least 0.153. Demonstrated here is that the current average level of political conflict (at −0.524, on average) does not play a significant role in mitigating the beneficial effect of financial development on the use of renewable energy sources. Although it is a substitute in the system. This may be due, in large part, to political unpredictability and violence within the context of African economies. This novel empirical insight explains why empirical findings from previous studies using panel selection of countries with different degrees of political risk and from the different multi-dimensional decomposition of financial development have remained inconclusive in their policy recommendations. For countries below the threshold of political conflict 0.002 to 1.396, political conflict plays a significant role in dragging financial development toward boosting renewable energy consumption in Africa.

Relying on the threshold political conflict of 0.002–1.396, we can assess the level of political conflict or stability of the countries in the panel that drags or boast the financial development. As shown in Appendix 4, the political situation of in Botswana, Mauritius, Cabo Verde, Namibia, Seychelles, Zambia, Sao Tome and Principe are within the range of the average threshold of 0.002–1.396, indicating that the political atmosphere in the region is fair enough to drive financial system towards boosting renewable energy. There is also an indication that further political stability in Gabon, Ghana and Benin could improve their financial system to drive renewable energy consumption. However, countries below the threshold are the vulnerable region, and their political situation constitutes a nuisance towards the financial system.

Gdp and population total are positive and significant determinants of renewable energy in Africa. A 1% increase in gdp and population increase renewable energy by 0.418%–0.550% and 0.068%–0.149%, respectively. This research's findings that a rise in Africa's population is associated with a rise in its GDP per capita are in line with previous empirical research. The model Spec [[1], [2], [3], [4], [5], [6], [7], [8], [9]] consistently shows a negative and statistically significant trade openness coefficient. According to the findings, free trade is a key factor in the development of renewable energy in Africa. This finding, however, runs counter to the spillover effects hypothesis, which has been supported by earlier research. These results are in line with new empirical reports from Africa. Many variables were cited in these studies as contributing to trade's negative impact in Africa. These included, but were not limited to, a high level of trade diversion, low levels of trade among African nations, and significant negative trade balances among African economies.

## 4 Summary and conclusion

In this article, we reexamine an old maxim about finance and renewable sources. This study looked at how political turmoil affects the connection between money and green power in Africa. According to the findings of this research, the level of political unrest in Africa is a critical factor in determining the amount of finance available to invest in renewable energy projects. Focusing on a panel of 46 African countries for the period 2010 to 2020 and based on the direct and indirect (interaction effect) strategy, the study examined the following questions: (i) what are the direct effect of various financial development indicators and political conflict explaining the level of renewable energy consumption in Africa? (ii) What is the moderating role of political conflict/level of financial development at which further improvement or deterioration could intensify/or drag renewable energy consumption in African economies? The IV-GMM parameter estimates are robust to cross-sectional dependence and allow for heterogeneous slope coefficients to prevent skewed parameter values. The key findings are summarized as follows:

• Financial development indicators and political conflict have a direct positive and negative effect on renewable energy consumption, which suggests that i.) An improved financial system could mobilize resources channeled toward adopting renewable energy technologies. ii.) The low average levels of political conflict indicators are one of the reasons why financial development hurts renewable energy adoption in some African regions.
• Political conflict in Africa considerably drags the favourable effect of financial development indicators on renewable energy consumption in some regions, but the weight is not strong enough to push it into a negative net effect.
• The interactive relationship between financial development and political conflict with the threshold level of political conflict is estimated at 0.002 to 1.396. This means that the political conflict below the threshold will trigger business environmental problems that will jeopardize the smooth-running financial system, reflecting the difficulty household and firms face in accessing credits to acquire renewable energy and vice versa.
• Excluding Botswana, Mauritius, Cabo Verde, Namibia, Seychelles, Zambia, Sao Tome and Principe, Gabon, Ghana, and Benin, all other countries in the sample have political conflict below the threshold of 0.002–1.396, which suggests the significant role of political conflict on finance-energy nexus in Africa.

Subtle recommendations to policymakers in the selected countries in the study sample follow findings from the econometric analysis conducted. Our research objective focused on ascertaining political conflict's role in Africa's finance-renewable energy nexus. African countries above the threshold should continue to strengthen proactive measures at the central, state and municipal levels to tackle any form of conflict, ethnic, religious, land-related or political, to preserve their stable political and business environment for foreign investment in the renewable energy sector. Furthermore, the focus can be extended beyond funding acquisition of military equipment to building human capacity in alternative dispute-resolution strategies that quell differences through dialogues and peace meetings. On the other hand, for countries below the threshold, the root cause of the continuous conflict must be ascertained, either political, religious and ethnic conflict or secession-based agitation. After that, specific and suitable strategies are deployed to various categories of conflict in sovereign states below the threshold. For areas with conflict emanating from the denial of human rights and other dividends of democracy, the military must pave the way to civilian administration to ensure that the fundamental rights enshrined in various International Charters and African Charters are guaranteed. In countries with session-induced conflict, governments should tackle the agenda and the demands of the secessionist using a series of legislation and executive orders. The above interventions should ensure equal political representation, a fair share of developmental projects, proper national security architecture representation and jobs in governmental agencies, departments and parastatals.

## Author contribution statement

Obumneke Bob Muoneke: conceived and designed the experiments, wrote the paper.

Kingsley Ikechukwu Okere: analyzed and interpreted the data, performed the experiments; Obiamaka Priscilla Egbo: contributed, materials, analysis tools or data; conceived and designed the experiments

## Funding statement

This research did not receive any specific grant from funding agencies in the public, commercial, or not-for-profit sectors.

## Data availability statement

Data will be made available on request.

## Declaration of interest’s statement

The authors declare no conflict of interest.

## Figures and Tables

**Table:** Definition of variables and data sources

variable	definition	sources
ren	Renewable energy consumption	WDI- (https://datacatalog.world bank.org/dataset/world-development-indicators)
Fin1-9	IMF multi-dimensional financial decomposition of financial index measures Financial development.	IMF (https://data.imf.org)
Confl	The political stability index is a subset of political governance that gauges how likely it is that the administration will be toppled due to factors such as domestic violence and terrorism. The scale runs from −2.5 weak to +2.5 strong in units.	WGI- https://datacatalog.world bank.org/dataset/worldwide-governance-indicators
loggdp	It stands for GDP per capita (constant 2010 US$)	WDI)
Logpop	It stands for Population, total	WDI
Int1-9	Interaction between financial development index and political conflict	-do-
Log Trade	Total trade (Export and Import % of GDP) measures Trade	WDI

**Appendix 2:** IV-GMM regression estimates based on Multi-dimensional approach to financial development

VARIABLES	1	2	3	4	5	6	7	8	9
	GL-1	GL-1	GL-1	GL-1	GL-1	GL-1	GL-1	GL-1	GL-1
Fin1	0.726***								
	(0.138)								
	[5.273]								
Int1	−0.520***								
	(0.145)								
	[-3.596]								
Fin2		0.638***							
		(0.096)							
		[6.681]							
Int2		−1.462***							
		(0.275)							
		[-5.310]							
Fin3			0.378***						
			(0.060)						
			[6.248]						
Int3			−1.134**						
			(0.449)						
			[-2.525]						
Fin4				0.120***					
				(0.083)					
				[2.440]					
Int4				−0.362***					
				(0.744)					
				[-3.487]					
Fin5					0.723***				
					(0.101)				
					[7.169]				
Int5					−3.997***				
					(1.026)				
					[-3.894]				
Fin6						0.013***			
						(0.062)			
						[3.200]			
Int6						−6.484***			
						(0.628)			
						[-5.152]			
Fin7							1.068***		
							(0.079)		
							[13.479]		
Int7							−6.821***		
							(0.378)		
							[-0.268]		
Fin8								0.034***	
								(0.054)	
								[5.628]	
Int8								−1.744***	
								(19.579)	
								[-8.910]	
Fin9									0.391***
									(0.120)
									[3.271]
Int9									−2.550***
									(43.682)
									[-5.839]
Confl	−0.092**	−0.041**	−0.031**	−0.016**	−0.034**	−0.011**	−0.039**	−0.015**	−0.022**
	(0.020)	(0.015)	(0.016)	(0.017)	(0.017)	(0.017)	(0.016)	(0.016)	(0.016)
	[-4.518]	[-2.750]	[-1.934]	[2.959]	[-2.067]	[−02674]	[-2.500]	[-3.931]	[-2.364]
logGdp	0.446***	0.418***	0.518***	0.535***	0.457***	0.550***	0.380***	0.520***	0.482***
	(0.045)	(0.047)	(0.044)	(0.043)	(0.044)	(0.048)	(0.037)	(0.044)	(0.047)
	[9.955]	[8.978]	[11.722]	[12.337]	[10.389]	[11.577]	[10.252]	[11.819]	[10.306]
logPoptol	0.122***	0.068***	0.149***	0.115***	0.139***	0.116***	0.137***	0.130***	0.141***
	(0.019)	(0.019)	(0.019)	(0.022)	(0.019)	(0.021)	(0.019)	(0.021)	(0.021)
	[6.608]	[3.675]	[7.754]	[5.169]	[7.363]	[5.406]	[7.151]	[6.180]	[6.803]
logTrade	−0.190***	−0.173***	−0.197***	−0.237***	−0.228***	−0.223***	−0.232***	−0.233***	−0.238***
	(0.052)	(0.044)	(0.062)	(0.059)	(0.057)	(0.058)	(0.055)	(0.059)	(0.058)
	[-3.684]	[-3.930]	[-3.203]	[-4.023]	[-3.990]	[-3.857]	[-4.234]	[-3.942]	[-4.099]
Constant	2.743***	2.973***	2.726***	3.089***	2.777***	3.045***	2.480***	2.879***	2.722***
	(0.157)	(0.176)	(0.172)	(0.192)	(0.161)	(0.190)	(0.150)	(0.183)	(0.179)
	[17.485]	[16.925]	[15.879]	[16.103]	[17.242]	[16.028]	[16.536]	[15.708]	[15.182]
***Diagnostic Test***									
Kleibergen-Paap rk LM statistic	5.793	5.843	5.893	5.943	5.993	6.043	6.093	6.143	6.193
P-val	0.122	0.119	0.116	0.113	0.11	0.107	0.104	0.101	0.098
Cragg-Donald Wald F statistic	425.546	500.853	329.144	322.112	273.911	225.710	177.509	129.308	81.107
Kleibergen-Paap rk Wald F statistic	56.722	107.477	411.635	546.858	124.314	901.771	109.227	126.684	144.140
Hansen J statistic	0.374	0.211	0.048	0.115	0.278	0.441	0.604	0.767	−0.13
P-val	0.5407	0.6462	0.7517	0.8572	0.9627	0.2682	0.1737	0.2792	0.3847
Time effect	Yes	Yes	Yes	Yes	Yes	Yes	Yes	Yes	Yes
Observations	460	460	460	460	460	460	460	460	460
R-squared	0.658	0.700	0.626	0.605	0.660	0.605	0.703	0.623	0.630

**Table:** Summary of marginal impact and threshold value of Political conflict

	Condition on Political Stability/crisis								
	Fin1	Fin2	Fin3	Fin4	Fin5	Fin6	Fin7	Fin8	Fin9
Mean- (Political Stability)	−0.524	−0.524	−0.524	−0.524	−0.524	−0.524	−0.524	−0.524	−0.524
Unconditional impact	0.726	0.638	0.378	0.12	0.723	0.013	1.068	0.034	0.391
Conditional impact	−0.52	−1.462	−1.134	−0.362	−3.997	−6.484	−6.821	−1.745	−2.551
Marginal impact	1.00	1.40	0.97	0.31	2.82	3.41	4.64	0.95	1.73
Threshold	1.396	0.436	0.333	0.331	0.181	0.002	0.157	0.019	0.153

**Appendix 4:** Countries above and below the threshold of Political Stability: Threshold Political Stability and Absence of Violence/Terrorism: 0.181 and 0.153 that trigger financial institution and market in Africa

Country	Mean
Botswana	1.040
Mauritius	0.883
Cabo Verde	0.785
Namibia	0.739
Seychelles	0.731
Zambia	0.233
Sao Tome and Principe	0.229
Gabon	0.079
Ghana	0.045
Benin	0.028
Gambia,	−0.021
Lesotho	−0.042
Equatorial Guinea	−0.058
Rwanda	−0.073
Malawi	−0.119
South Africa	−0.145
Senegal	−0.150
Sierra Leone	−0.154
Comoros	−0.268
Tanzania	−0.322
Eswatini	−0.348
Angola	−0.374
Mozambique	−0.441
Liberia	−0.452
Togo	−0.479
Congo, Rep.	−0.512
Madagascar	−0.535
Guinea-Bissau	−0.678
Eritrea	−0.770
Zimbabwe	−0.810
Mauritania	−0.811
Uganda	−0.811
Burkina Faso	−0.827
Guinea	−0.948
Cameroon	−1.017
Cote d'Ivoire	−1.102
Kenya	−1.200
Niger	−1.232
Chad	−1.289
Ethiopia	−1.508
Burundi	−1.629
Mali	−1.636
Nigeria	−2.011
Central African Republic	−2.070
Congo, Dem. Rep.	−2.090
South Sudan	−2.138

## Appendix 5

Angola, Benin, Botswana, Burkina Faso, Burundi, Cape Verde, Cameroon, Central African Rep, Chad, Comoros, Con-Dem Rep, Côte d'Ivoire, Equatorial Guinea, Eritrea, Eswatini, Ethiopia, Gabon, Gambia, Ghana, Guinea, Guinea-Bissau, Kenya, Lesotho, Liberia, Madagascar, Malawi, Mali, Mauritius, Mozambique, Namibia, Niger, Nigeria, Rwanda, Sao Tome and Principe, Senegal, Seychelles, Sierra Leone, South Africa, South Sudan, Tanzania, Togo, Uganda, Zambia and Zimbabwe.

## Footnotes

1 It also indicates the moderating role of political conflict on finance-energy nexus.
